# Supplementary material for: Iron rescues glucose-mediated photosynthesis repression during lipid accumulation in the green alga Chromochloris zofingiensis
Source: Nat Commun. 2024 Jul 18;15:6046. doi: 10.1038/s41467-024-50170-x (PMC11258321; doi:10.1038/s41467-024-50170-x)
Supplement: Supplementary file 3 — Description of Additional Supplementary Files [file 41467_2024_50170_MOESM3_ESM.pdf]

Description of Additional Supplementary Files

Supplementary Data 1. Chlorophyll fluorescence and growth curves of *Chromochloris zofingiensis* after iron and glucose treatments.

Supplementary Data 2. Collection of light and transmission electron microscopy images of *Chromochloris zofingiensis* under different trophic states induced by iron and glucose.

Supplementary Data 3. Proteomics of twelve iron and glucose conditions in WT and *hxk1* mutants. Raw abundance data, linear modeling group assignments, and results of GO term enrichment analysis are included.

Supplementary Data 4. Oxygen evolution and consumption and chlorophyll concentrations of twelve iron and glucose conditions in WT and *hxk1* mutants.

Supplementary Data 5. Proteomic enrichment of photosynthetic and heterotrophic proteins in *Chromochloris zofingiensis,* including conserved co-regulation with photosynthesis across species and GO enrichment results.

Supplementary Data 6. Regulation of the photosynthetic and respiratory electron transport chain across iron conditions and iron and glucose regulation of known iron transporters.

Supplementary Data 7. Proteomic enrichment of lipid accumulation players, including overlap with lipid droplet proteome and GO enrichment results.

Supplementary Data 8. Regulation of iron containing ferredoxins and fatty acid desaturases to iron and glucose.

Supplementary Data 9. Updated *Chromochloris zofingiensis* protein annotations and predicted *Arabidopsis thaliana* and *Chlamydomonas reinhardtii* orthologs.
