# Supplementary material for: Iron rescues glucose-mediated photosynthesis repression during lipid accumulation in the green alga Chromochloris zofingiensis
Source: Nat Commun. 2024 Jul 18;15:6046. doi: 10.1038/s41467-024-50170-x (PMC11258321; doi:10.1038/s41467-024-50170-x)
Supplement: Supplementary file 5 — Supplementary Data 2 [file 41467_2024_50170_MOESM5_ESM.zip › Supplementary_Dataset_2_Microscropy_and_TLC_Images/Light Microscopy Images/ReadME.rtf]

Due to their file size, the original jpegs of the light microscopy imagesCan be found at https://osf.io/r8dbe/  under the folder“Original Light Microscopy Photographs”
