# Supplementary figures and images for: Iron rescues glucose-mediated photosynthesis repression during lipid accumulation in the green alga Chromochloris zofingiensis
Source: Nat Commun. 2024 Jul 18;15:6046. doi: 10.1038/s41467-024-50170-x (PMC11258321; doi:10.1038/s41467-024-50170-x)

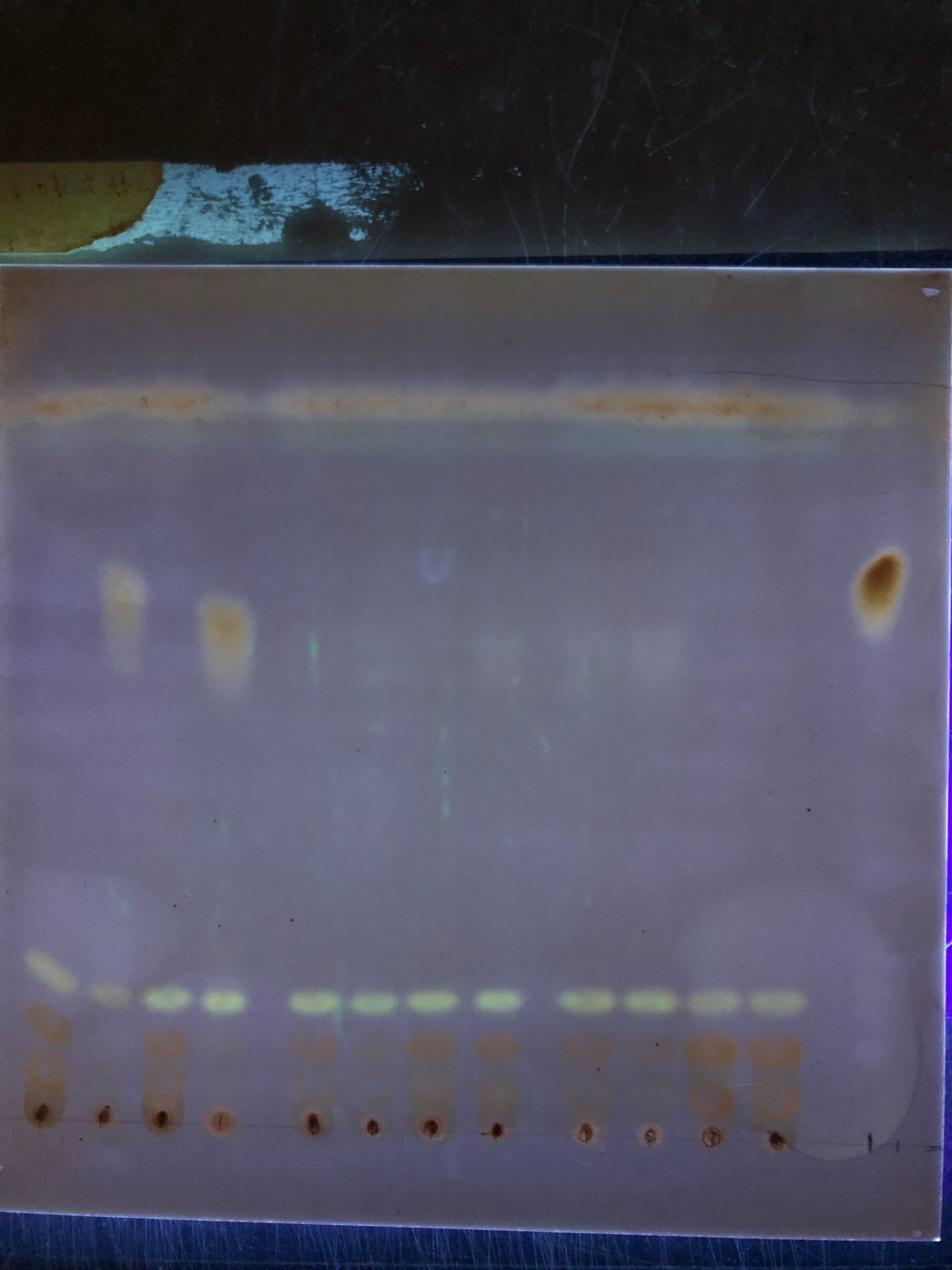

Supplement: Supplementary file 5 — Supplementary Data 2 [file 41467_2024_50170_MOESM5_ESM.zip › Supplementary_Dataset_2_Microscropy_and_TLC_Images/TLC_Plates_Uncropped/Fig.7a_Uncropped_TLC_UV.jpg]

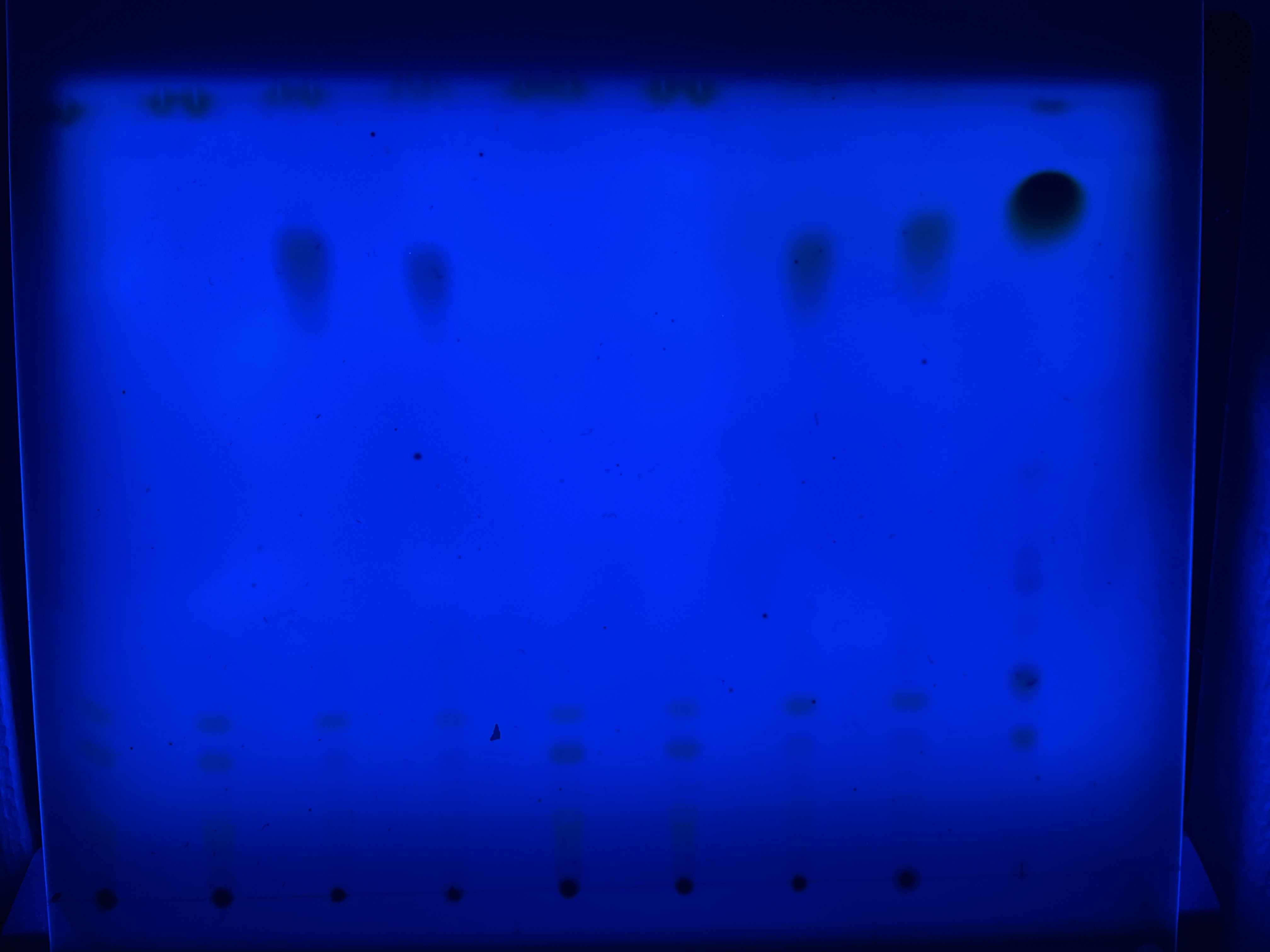

Supplement: Supplementary file 5 — Supplementary Data 2 [file 41467_2024_50170_MOESM5_ESM.zip › Supplementary_Dataset_2_Microscropy_and_TLC_Images/TLC_Plates_Uncropped/Fig.2e.Uncropped_TLC_UV_withreplicates.jpg]

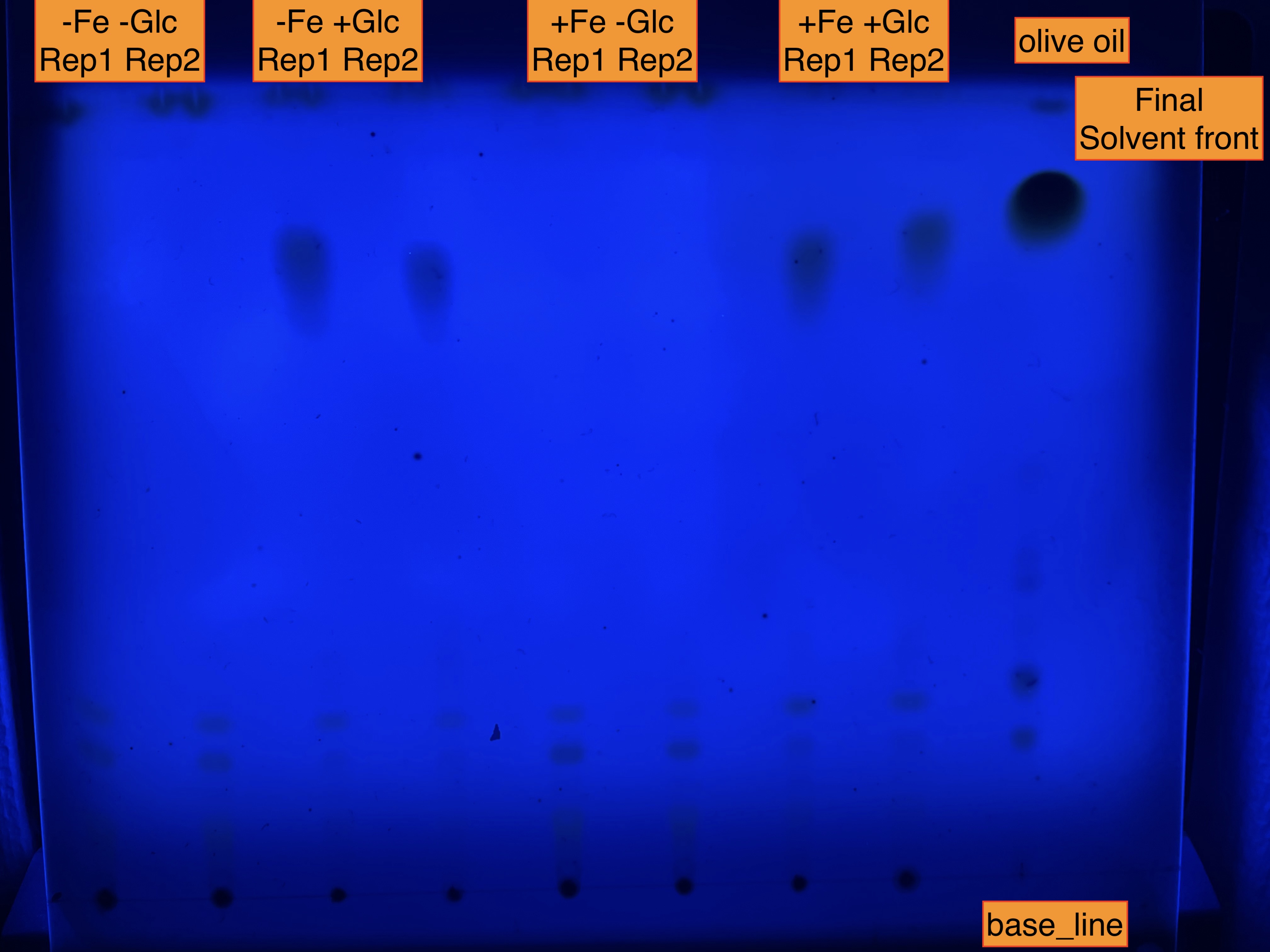

Supplement: Supplementary file 5 — Supplementary Data 2 [file 41467_2024_50170_MOESM5_ESM.zip › Supplementary_Dataset_2_Microscropy_and_TLC_Images/TLC_Plates_Uncropped/Fig.2e.Uncropped_TLC_UV_withreplicates Annotated.jpeg]

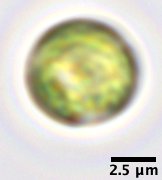

Supplement: Supplementary file 5 — Supplementary Data 2 [file 41467_2024_50170_MOESM5_ESM.zip › Supplementary_Dataset_2_Microscropy_and_TLC_Images/Light Microscopy Images/Cropped and Scale Bar/20210501_D641_+Fe-Glc.jpg]

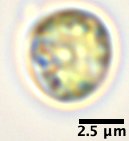

Supplement: Supplementary file 5 — Supplementary Data 2 [file 41467_2024_50170_MOESM5_ESM.zip › Supplementary_Dataset_2_Microscropy_and_TLC_Images/Light Microscopy Images/Cropped and Scale Bar/20210429_D918-1_-Fe-Glc.jpg]

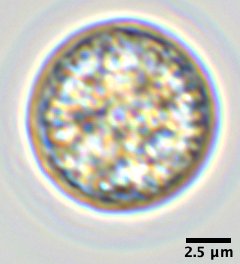

Supplement: Supplementary file 5 — Supplementary Data 2 [file 41467_2024_50170_MOESM5_ESM.zip › Supplementary_Dataset_2_Microscropy_and_TLC_Images/Light Microscopy Images/Cropped and Scale Bar/20210429_WT05_-Fe+Glc.jpg]

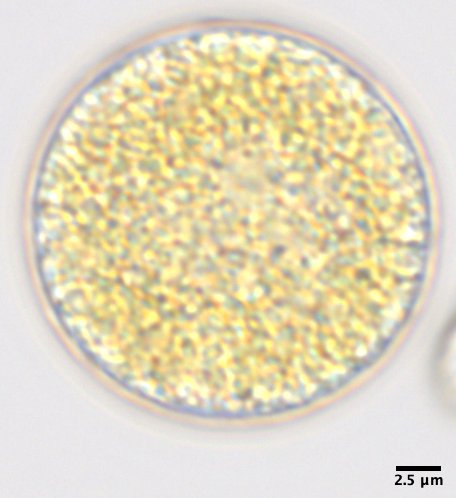

Supplement: Supplementary file 5 — Supplementary Data 2 [file 41467_2024_50170_MOESM5_ESM.zip › Supplementary_Dataset_2_Microscropy_and_TLC_Images/Light Microscopy Images/Cropped and Scale Bar/20210429_WT15_+Fe+Glc.jpg]

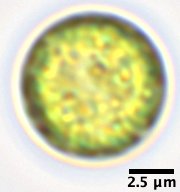

Supplement: Supplementary file 5 — Supplementary Data 2 [file 41467_2024_50170_MOESM5_ESM.zip › Supplementary_Dataset_2_Microscropy_and_TLC_Images/Light Microscopy Images/Cropped and Scale Bar/20210429_D927-3_+Fe-Glc.jpg]

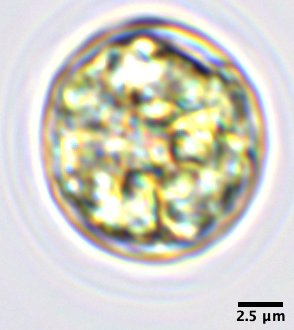

Supplement: Supplementary file 5 — Supplementary Data 2 [file 41467_2024_50170_MOESM5_ESM.zip › Supplementary_Dataset_2_Microscropy_and_TLC_Images/Light Microscopy Images/Cropped and Scale Bar/20210501_D638_-Fe+Glc.jpg]

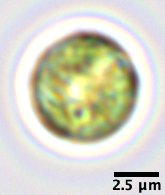

Supplement: Supplementary file 5 — Supplementary Data 2 [file 41467_2024_50170_MOESM5_ESM.zip › Supplementary_Dataset_2_Microscropy_and_TLC_Images/Light Microscopy Images/Cropped and Scale Bar/20210429_WT09_+Fe-Glc.jpg]

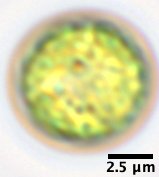

Supplement: Supplementary file 5 — Supplementary Data 2 [file 41467_2024_50170_MOESM5_ESM.zip › Supplementary_Dataset_2_Microscropy_and_TLC_Images/Light Microscopy Images/Cropped and Scale Bar/20210429_D9_27-3_+Fe-Glc.jpg]

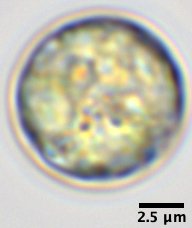

Supplement: Supplementary file 5 — Supplementary Data 2 [file 41467_2024_50170_MOESM5_ESM.zip › Supplementary_Dataset_2_Microscropy_and_TLC_Images/Light Microscopy Images/Cropped and Scale Bar/20210429_D921-3_-Fe+Glc.jpg]

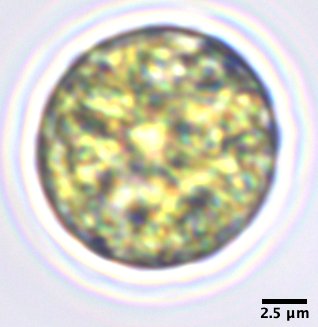

Supplement: Supplementary file 5 — Supplementary Data 2 [file 41467_2024_50170_MOESM5_ESM.zip › Supplementary_Dataset_2_Microscropy_and_TLC_Images/Light Microscopy Images/Cropped and Scale Bar/20210501_D645-3_+Fe+Glc.jpg]

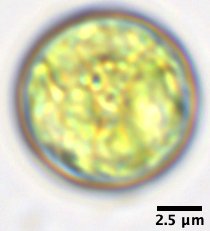

Supplement: Supplementary file 5 — Supplementary Data 2 [file 41467_2024_50170_MOESM5_ESM.zip › Supplementary_Dataset_2_Microscropy_and_TLC_Images/Light Microscopy Images/Cropped and Scale Bar/20210429_D931-3_+Fe+Glc.jpg]

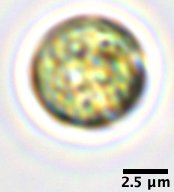

Supplement: Supplementary file 5 — Supplementary Data 2 [file 41467_2024_50170_MOESM5_ESM.zip › Supplementary_Dataset_2_Microscropy_and_TLC_Images/Light Microscopy Images/Cropped and Scale Bar/20210501_D633_-Fe-Glc.jpg]

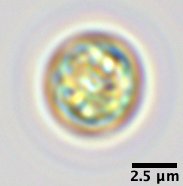

Supplement: Supplementary file 5 — Supplementary Data 2 [file 41467_2024_50170_MOESM5_ESM.zip › Supplementary_Dataset_2_Microscropy_and_TLC_Images/Light Microscopy Images/Cropped and Scale Bar/20210429_WT01_-Fe-Glc.jpg]

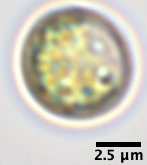

Supplement: Supplementary file 5 — Supplementary Data 2 [file 41467_2024_50170_MOESM5_ESM.zip › Supplementary_Dataset_2_Microscropy_and_TLC_Images/Light Microscopy Images/Cropped and Scale Bar/20210429_D919-1_-Fe-Glc.jpg]

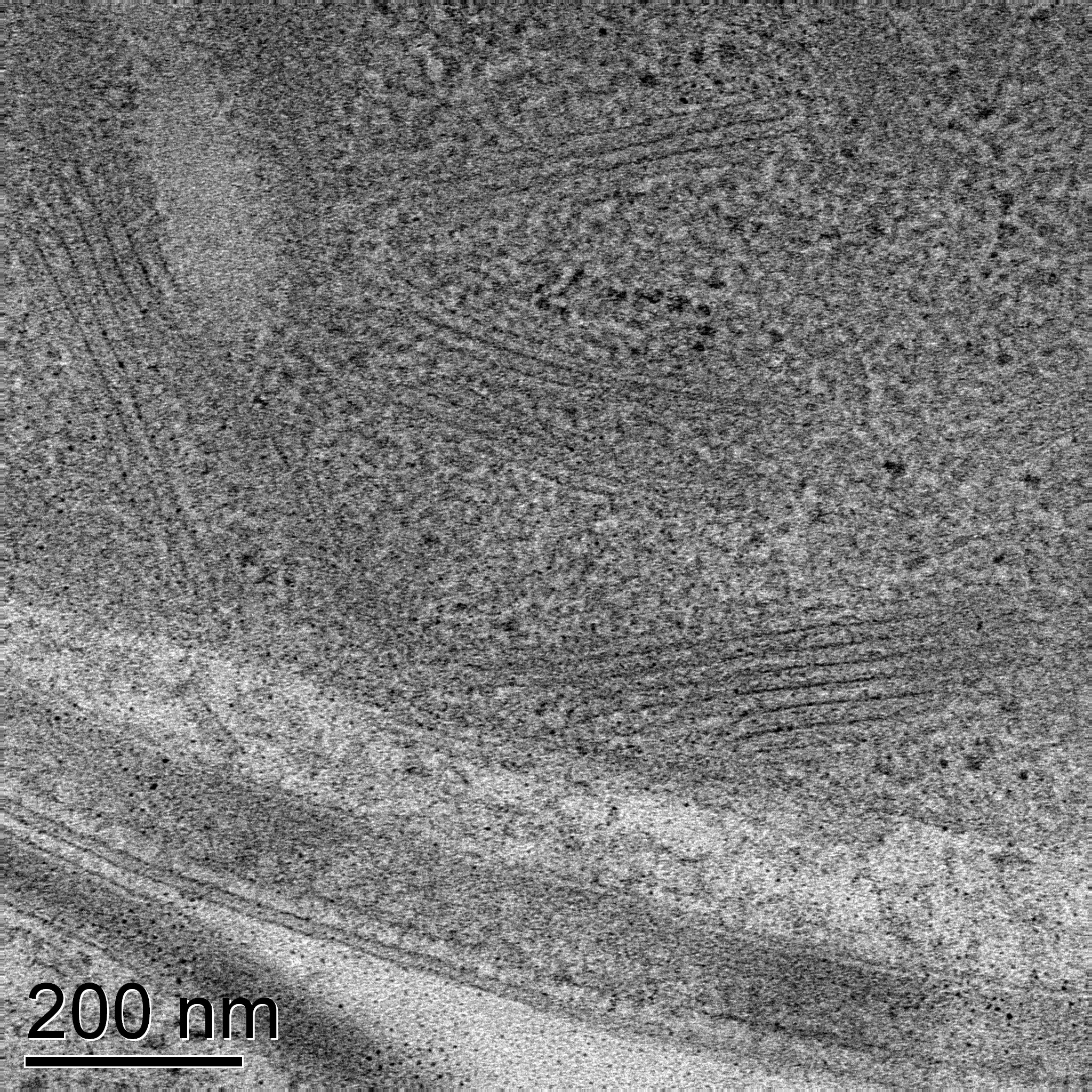

Supplement: Supplementary file 5 — Supplementary Data 2 [file 41467_2024_50170_MOESM5_ESM.zip › Supplementary_Dataset_2_Microscropy_and_TLC_Images/TEM_Images/+Fe-Glc/B-wt25s-12.jpg]

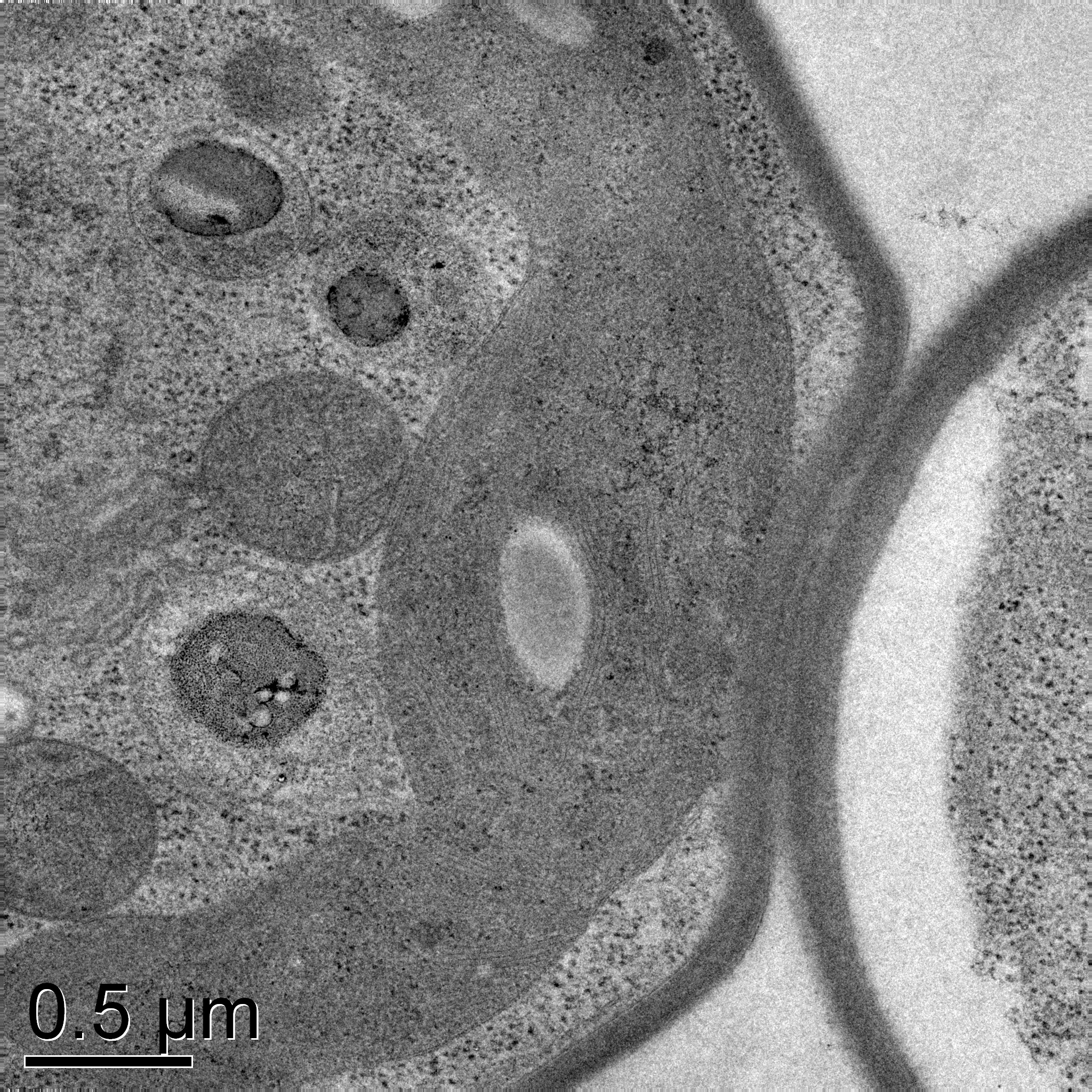

Supplement: Supplementary file 5 — Supplementary Data 2 [file 41467_2024_50170_MOESM5_ESM.zip › Supplementary_Dataset_2_Microscropy_and_TLC_Images/TEM_Images/+Fe-Glc/B-wt25s-26.jpg]

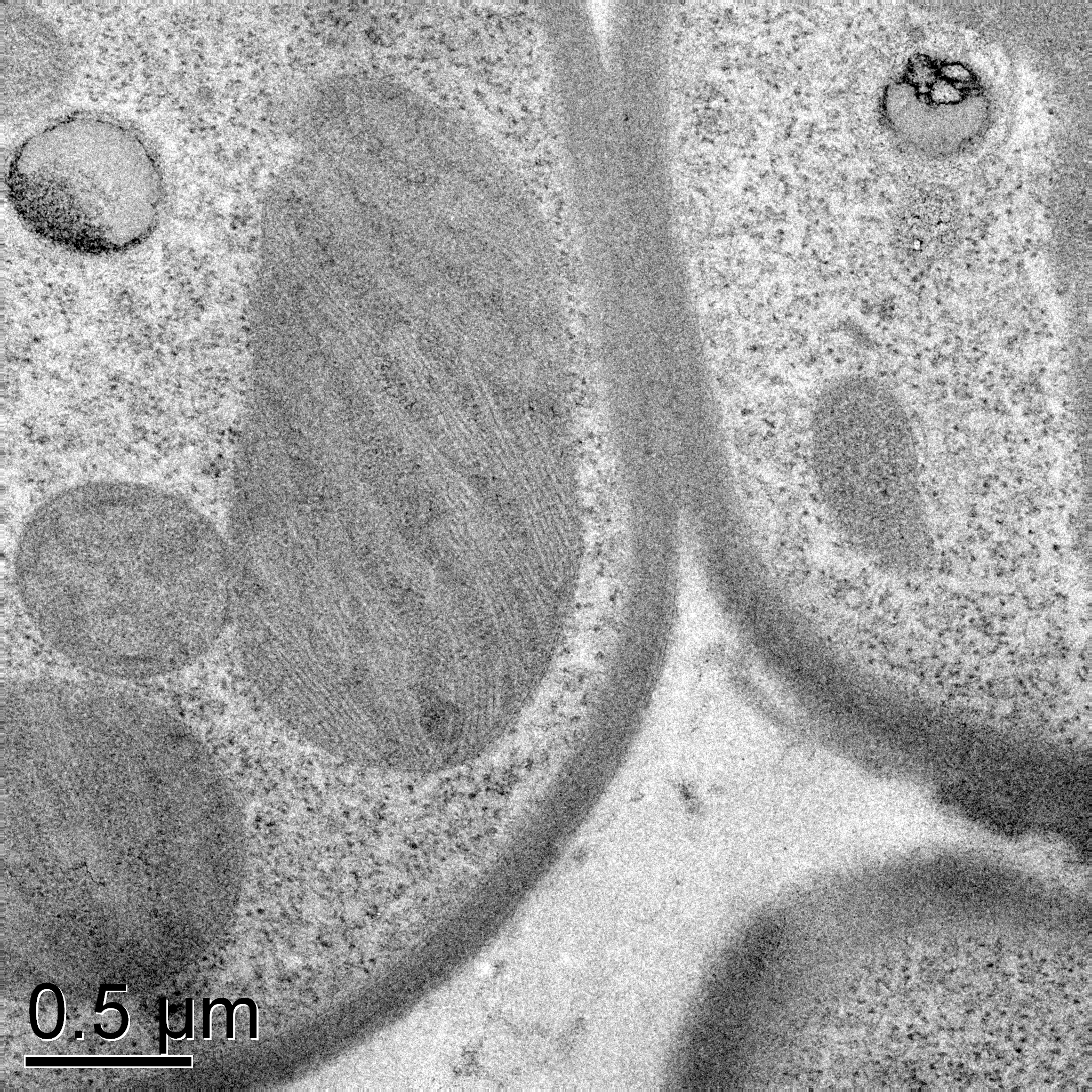

Supplement: Supplementary file 5 — Supplementary Data 2 [file 41467_2024_50170_MOESM5_ESM.zip › Supplementary_Dataset_2_Microscropy_and_TLC_Images/TEM_Images/+Fe-Glc/B-wt25s-5.jpg]

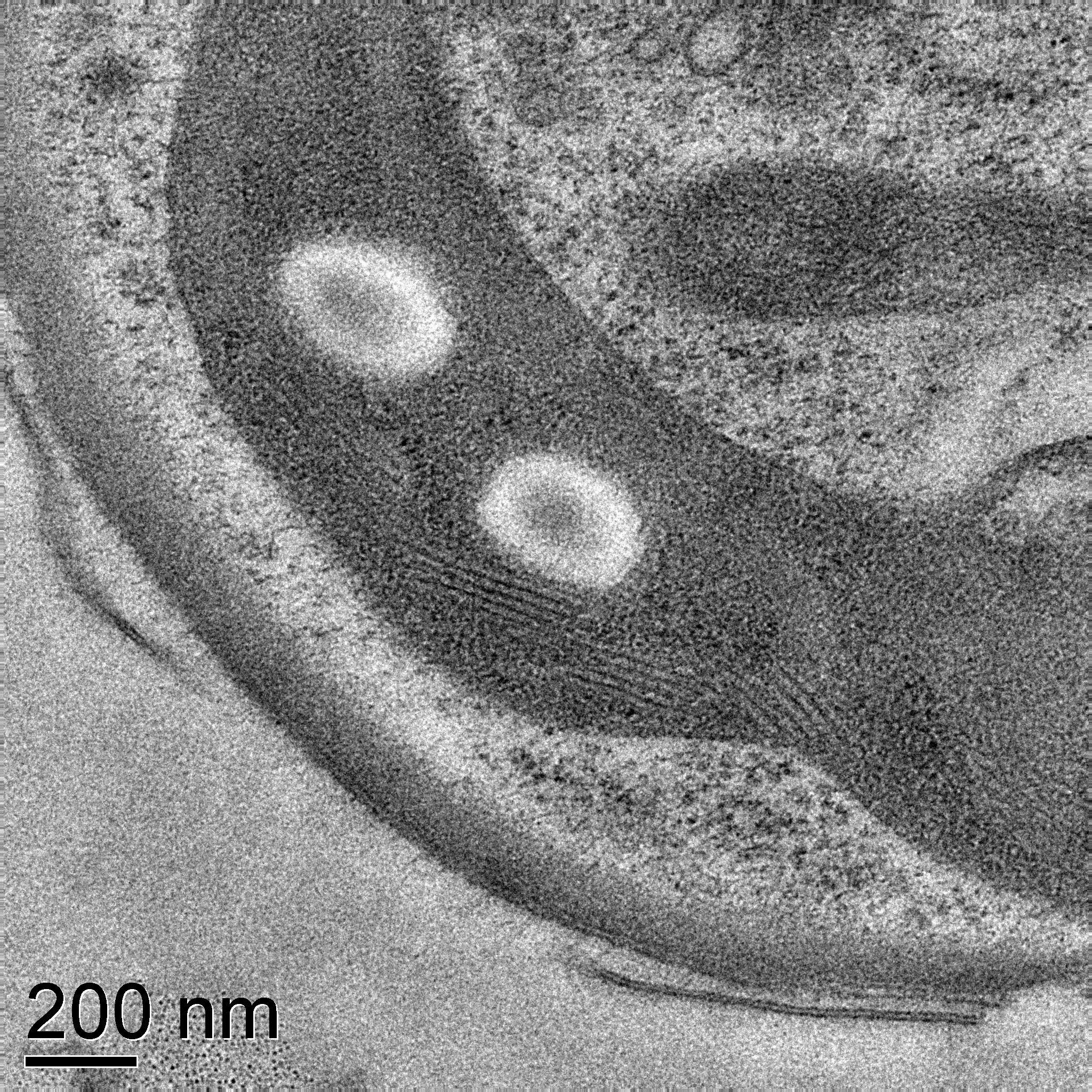

Supplement: Supplementary file 5 — Supplementary Data 2 [file 41467_2024_50170_MOESM5_ESM.zip › Supplementary_Dataset_2_Microscropy_and_TLC_Images/TEM_Images/-Fe-Glc/A-wt17_s-23.jpg]

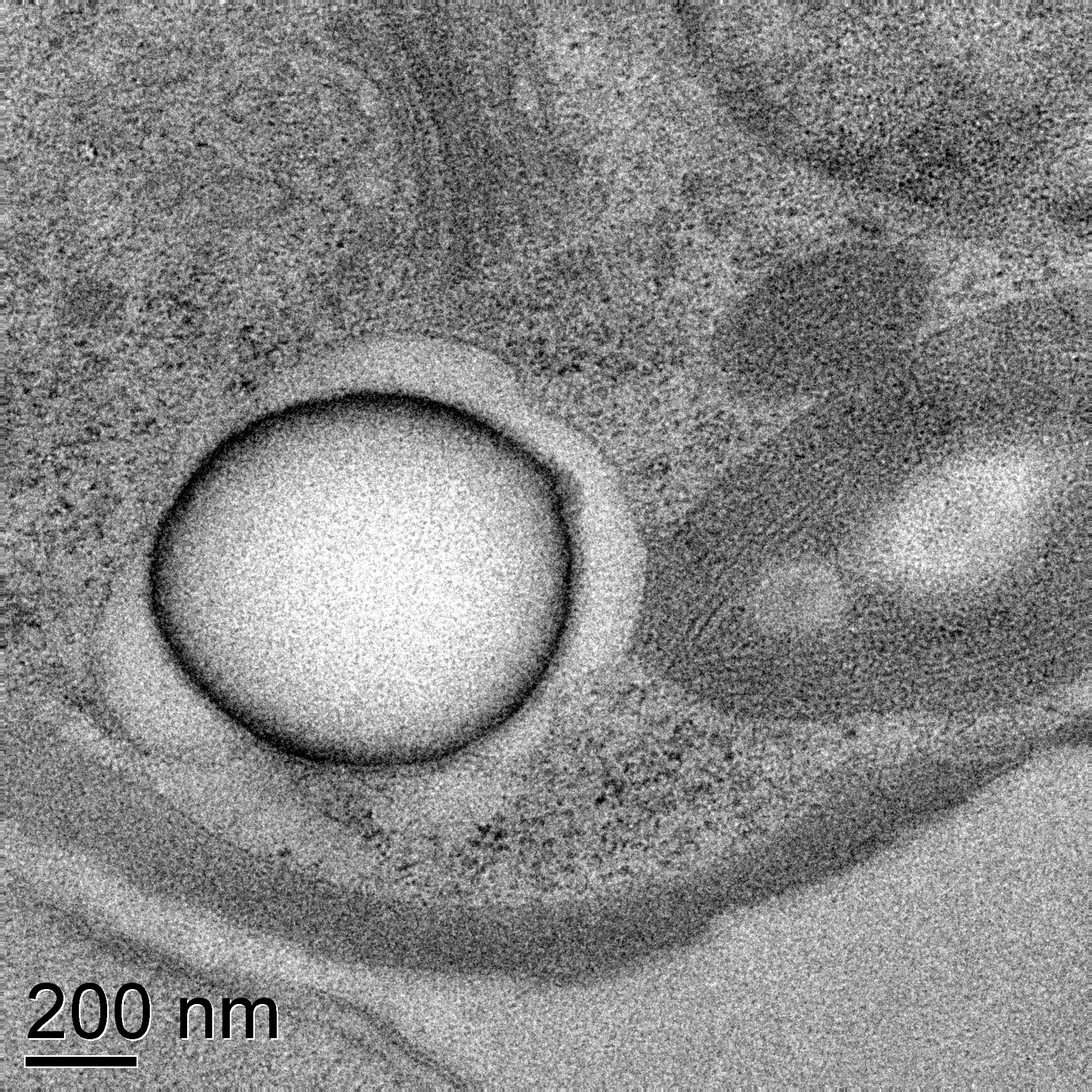

Supplement: Supplementary file 5 — Supplementary Data 2 [file 41467_2024_50170_MOESM5_ESM.zip › Supplementary_Dataset_2_Microscropy_and_TLC_Images/TEM_Images/-Fe-Glc/A-wt17_s-34.jpg]

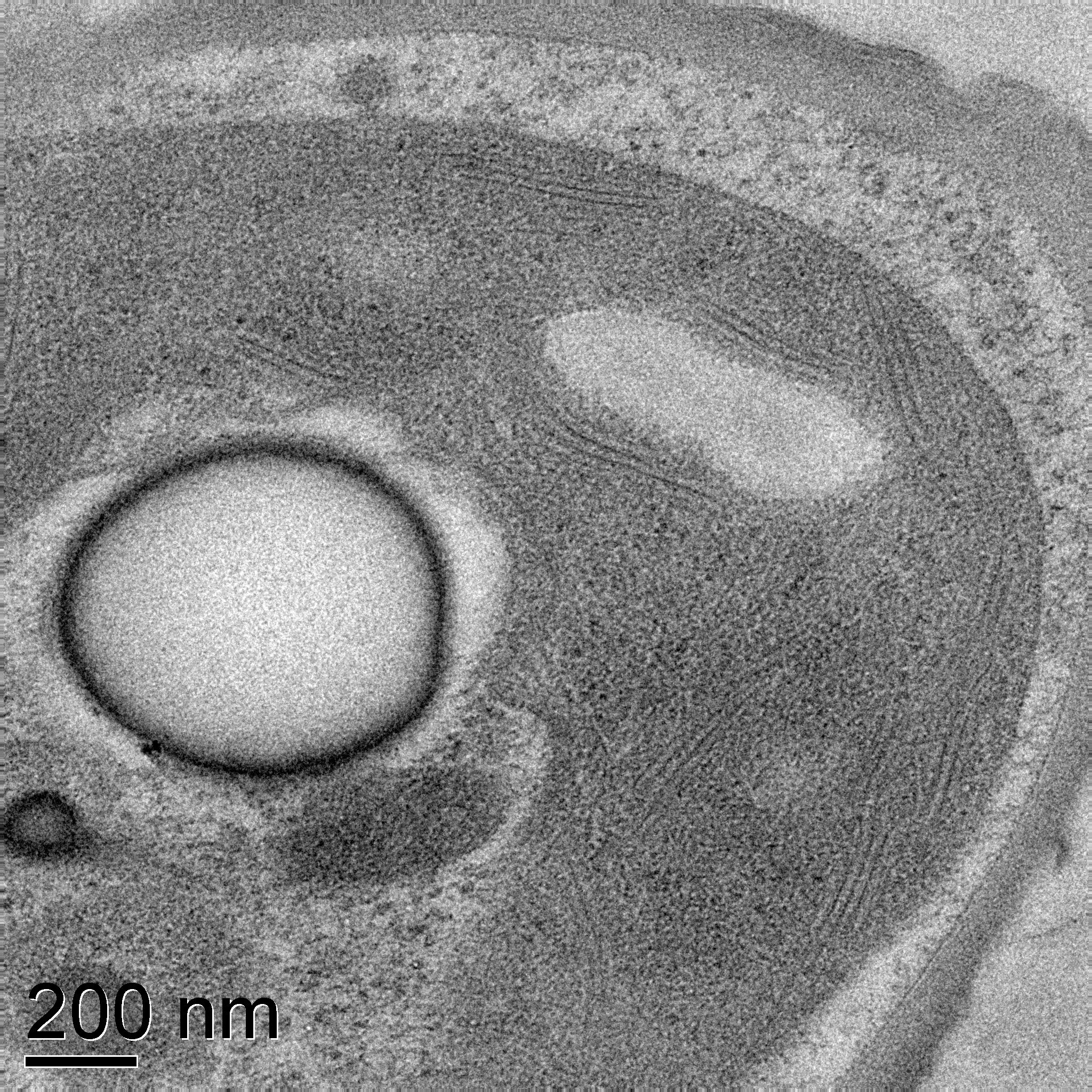

Supplement: Supplementary file 5 — Supplementary Data 2 [file 41467_2024_50170_MOESM5_ESM.zip › Supplementary_Dataset_2_Microscropy_and_TLC_Images/TEM_Images/-Fe-Glc/A-wt17_s-17.jpg]

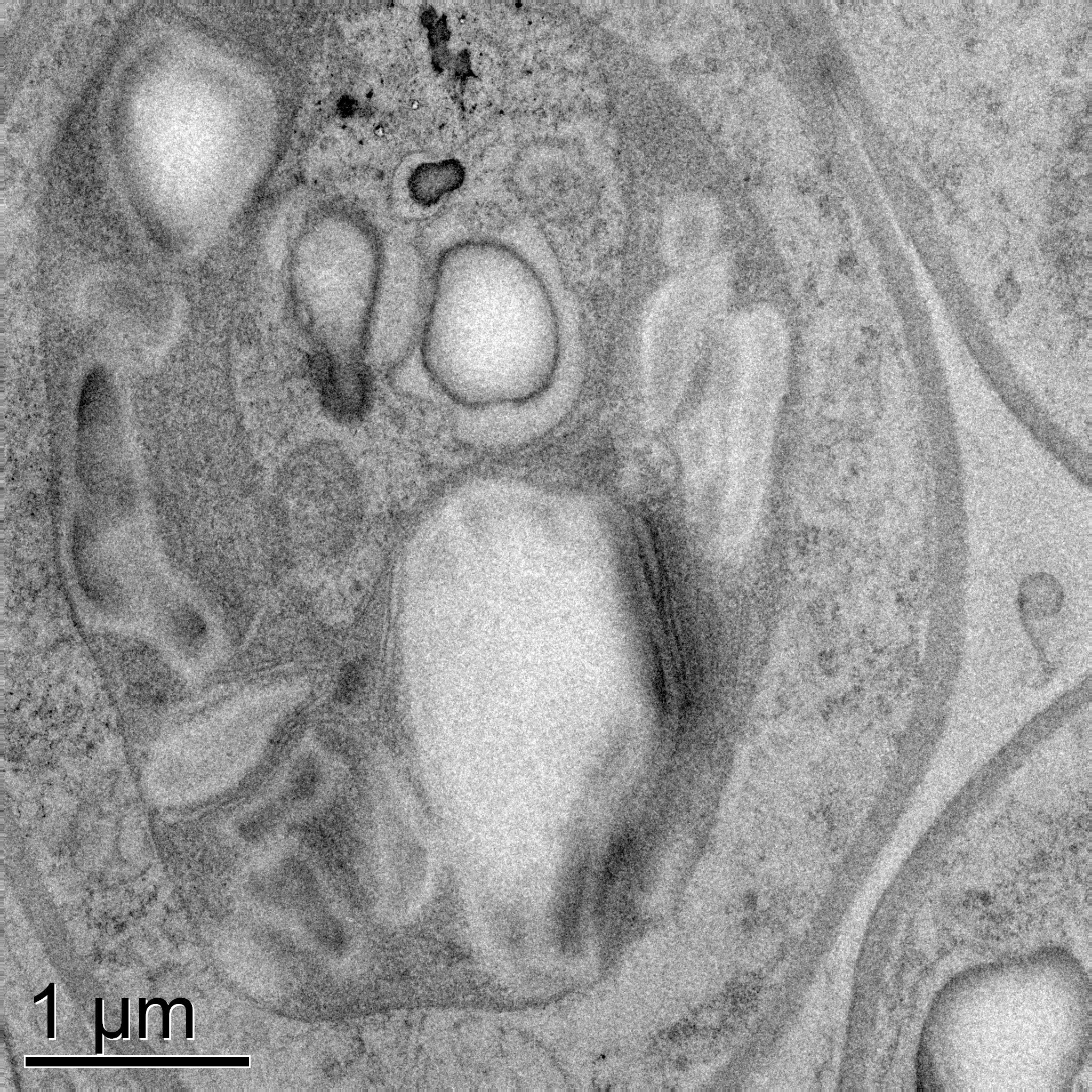

Supplement: Supplementary file 5 — Supplementary Data 2 [file 41467_2024_50170_MOESM5_ESM.zip › Supplementary_Dataset_2_Microscropy_and_TLC_Images/TEM_Images/+Fe+Glc/D-wt29_+Gl+Fe-17.jpg]

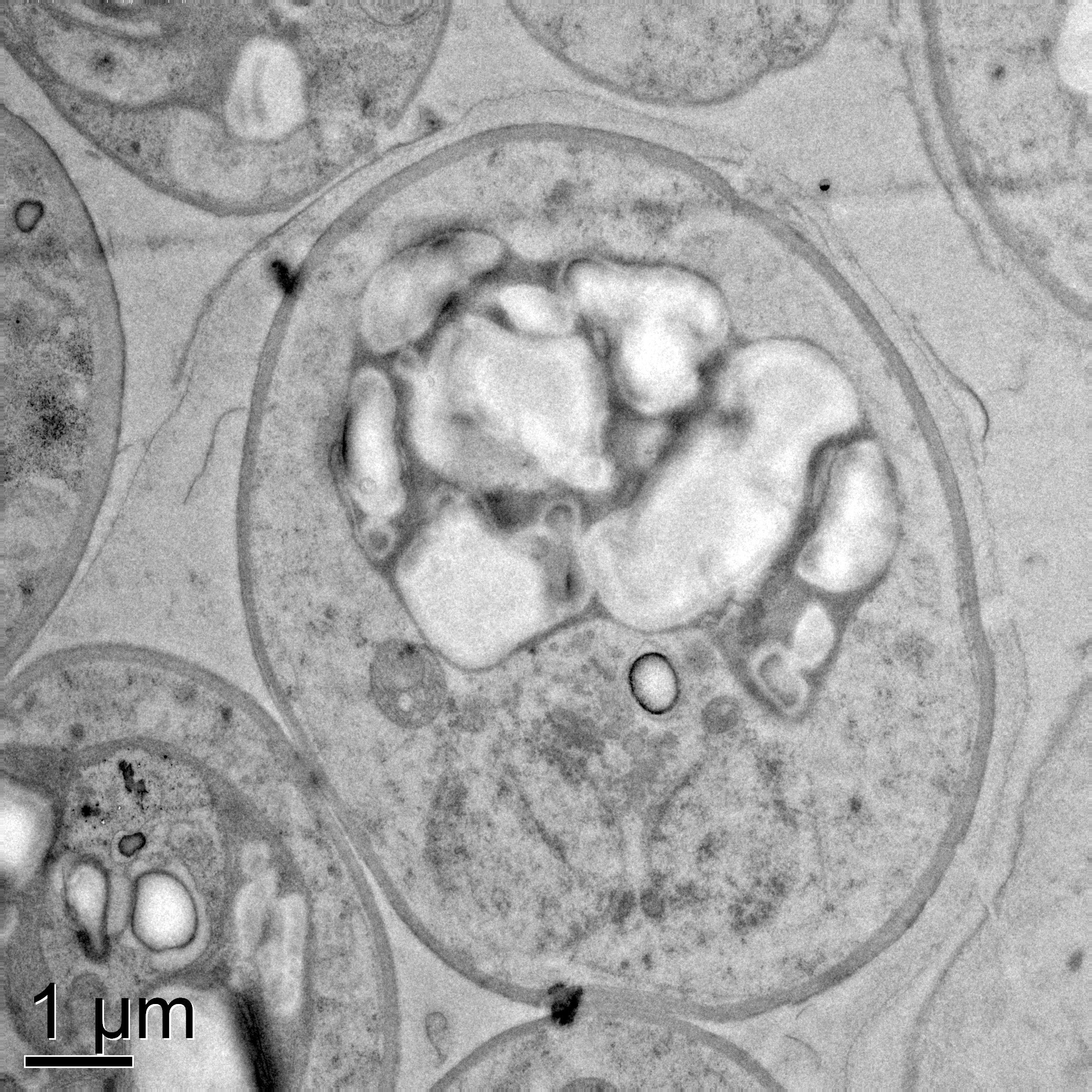

Supplement: Supplementary file 5 — Supplementary Data 2 [file 41467_2024_50170_MOESM5_ESM.zip › Supplementary_Dataset_2_Microscropy_and_TLC_Images/TEM_Images/+Fe+Glc/D-wt29_+Gl+Fe-7.jpg]

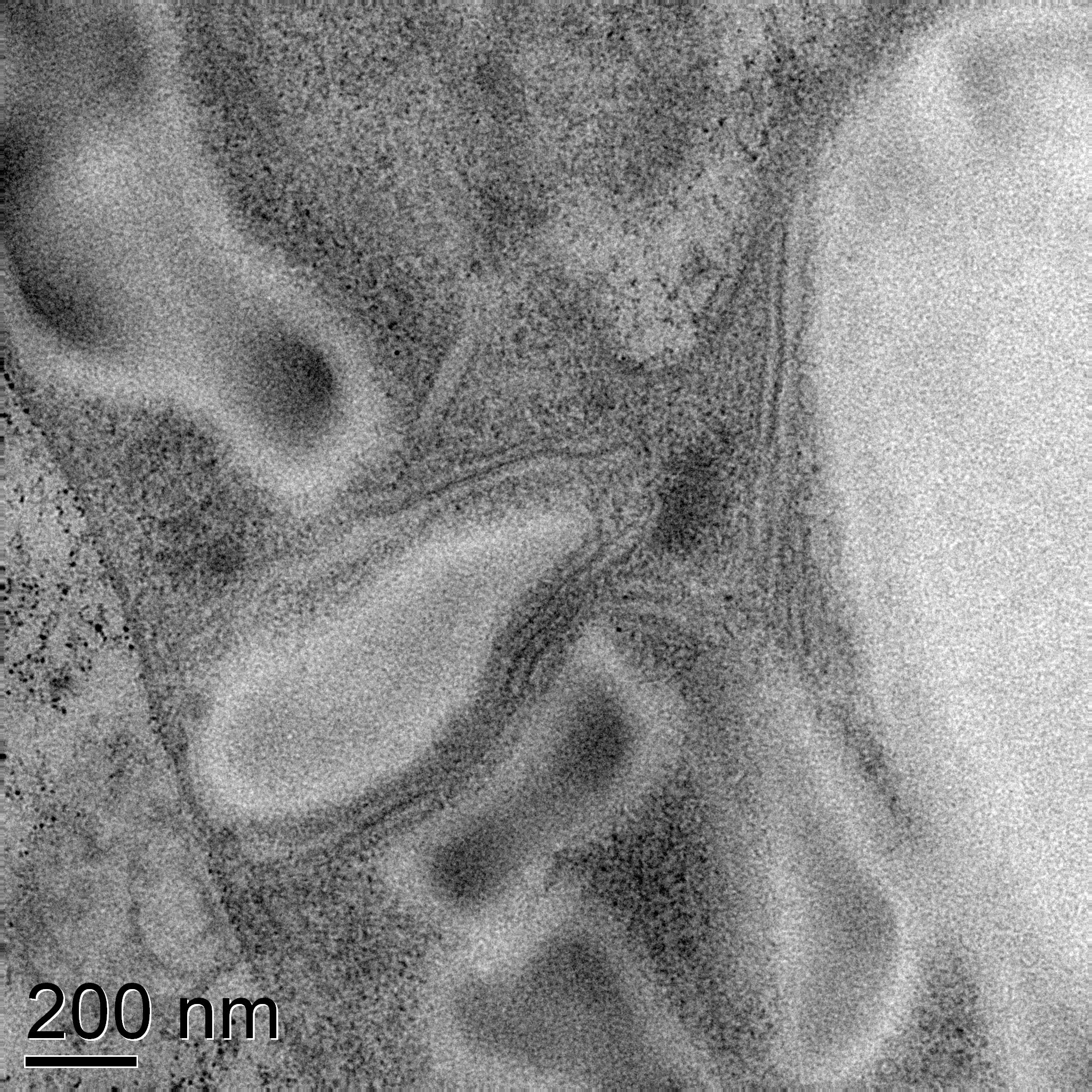

Supplement: Supplementary file 5 — Supplementary Data 2 [file 41467_2024_50170_MOESM5_ESM.zip › Supplementary_Dataset_2_Microscropy_and_TLC_Images/TEM_Images/+Fe+Glc/D-wt29_+Gl+Fe-21.jpg]

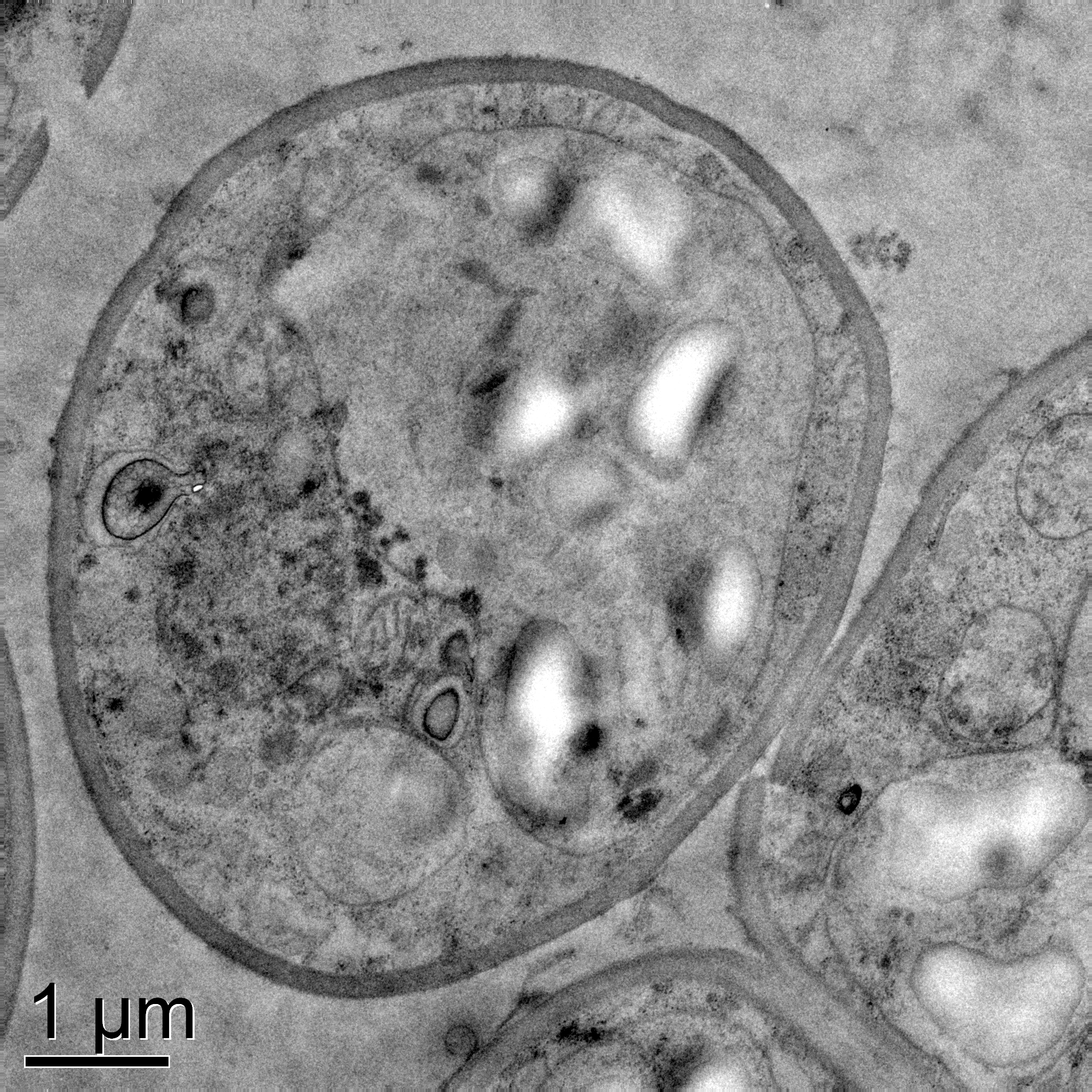

Supplement: Supplementary file 5 — Supplementary Data 2 [file 41467_2024_50170_MOESM5_ESM.zip › Supplementary_Dataset_2_Microscropy_and_TLC_Images/TEM_Images/-Fe+Glc/C-wt21_-Fe-5.jpg]

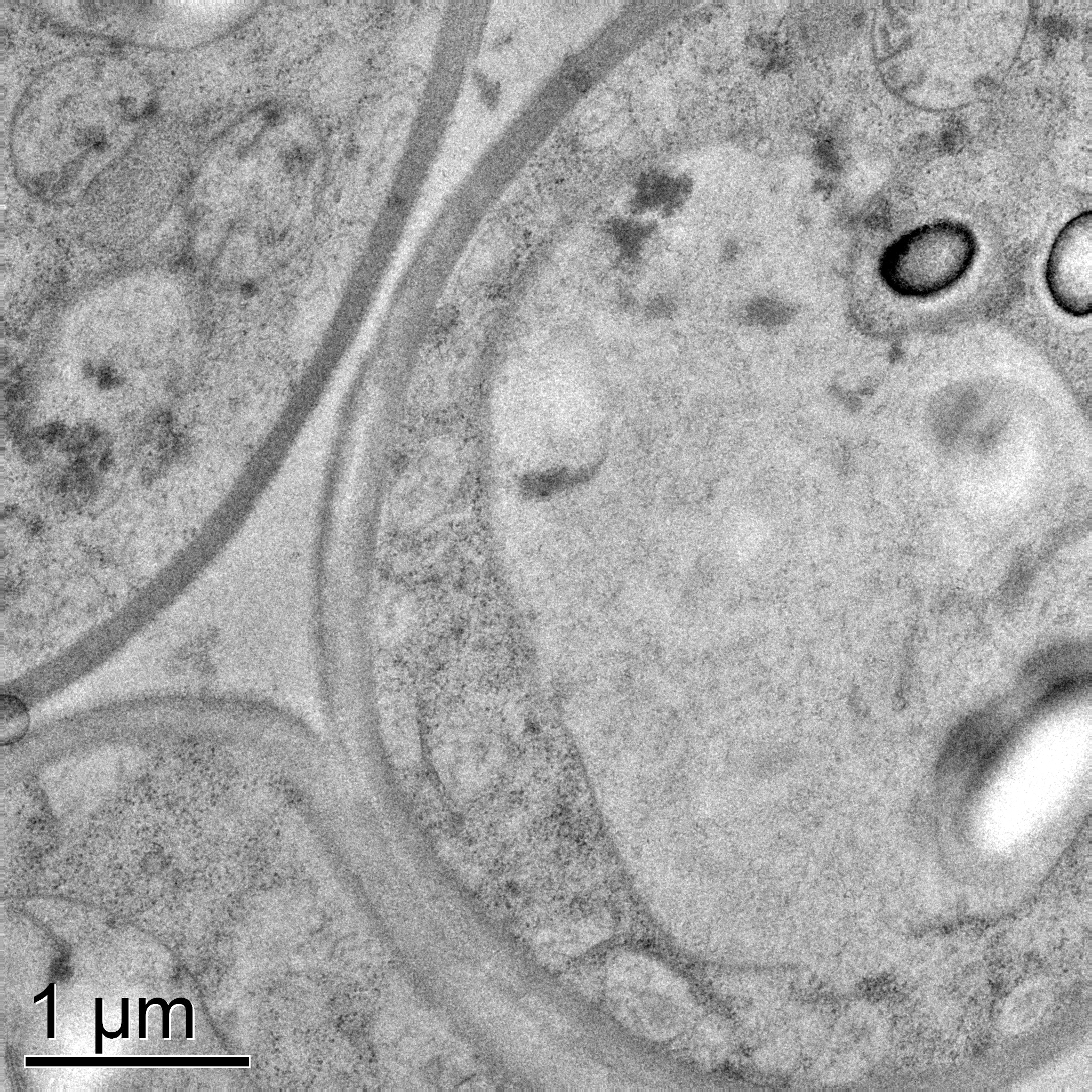

Supplement: Supplementary file 5 — Supplementary Data 2 [file 41467_2024_50170_MOESM5_ESM.zip › Supplementary_Dataset_2_Microscropy_and_TLC_Images/TEM_Images/-Fe+Glc/C-wt21_-Fe-50.jpg]

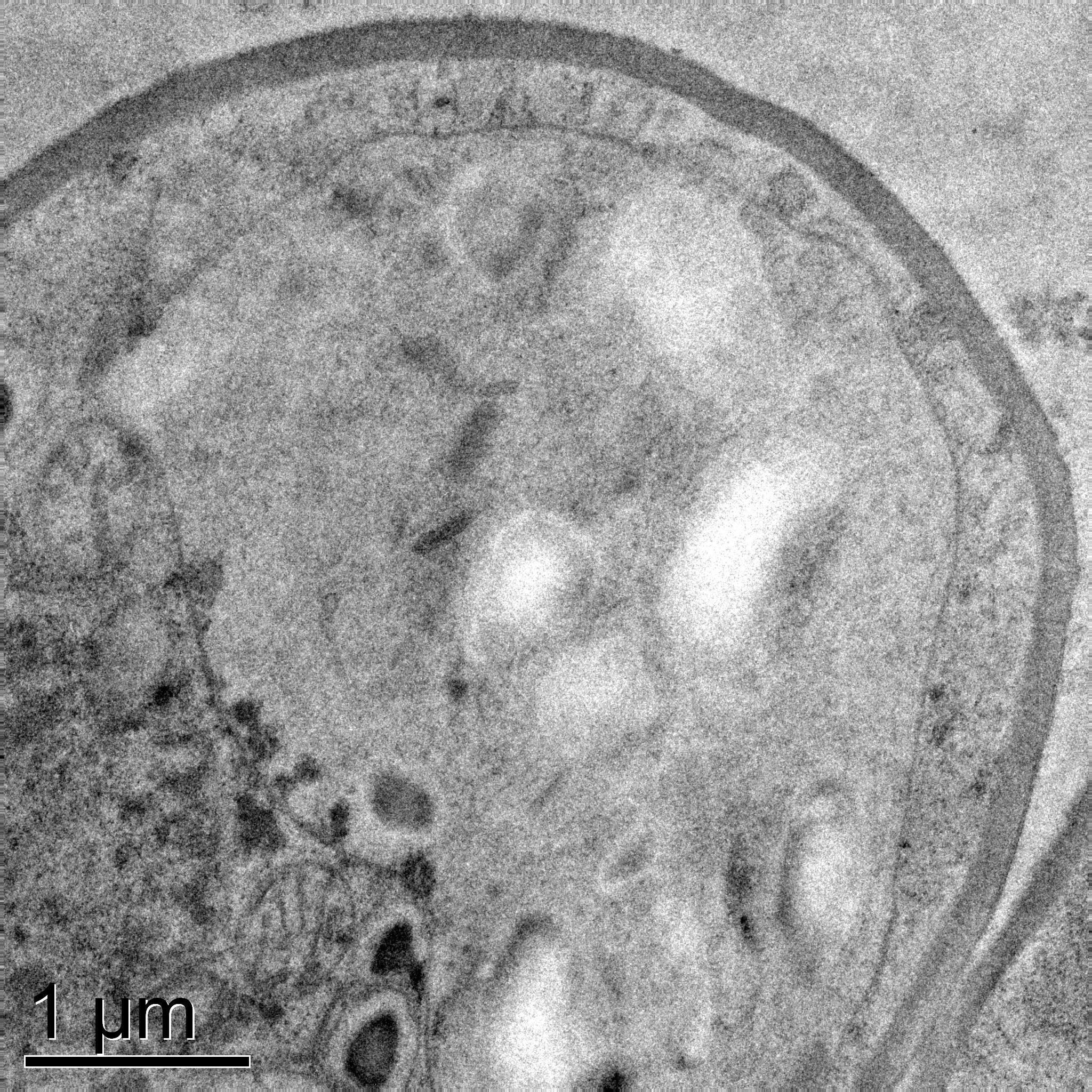

Supplement: Supplementary file 5 — Supplementary Data 2 [file 41467_2024_50170_MOESM5_ESM.zip › Supplementary_Dataset_2_Microscropy_and_TLC_Images/TEM_Images/-Fe+Glc/C-wt21_-Fe-33.jpg]
