# Supplementary material for: Iron rescues glucose-mediated photosynthesis repression during lipid accumulation in the green alga Chromochloris zofingiensis
Source: Nat Commun. 2024 Jul 18;15:6046. doi: 10.1038/s41467-024-50170-x (PMC11258321; doi:10.1038/s41467-024-50170-x)
Supplement: Supplementary file 13 — Reporting Summary [file 41467_2024_50170_MOESM13_ESM.pdf]

nature portfolio

Corresponding author(s): Melissa S. Roth, Krishna K. Niyogi

Last updated by author(s): Aug 17, 2023

Reporting Summary

Nature Portfolio wishes to improve the reproducibility of the work that we publish. This form provides structure for consistency and transparency in reporting. For further information on Nature Portfolio policies, see our Editorial Policies and the Editorial Policy Checklist.

Statistics

For all statistical analyses, confirm that the following items are present in the figure legend, table legend, main text, or Methods section.

n/a	Confirmed	
☐	☒	The exact sample size (*n*) for each experimental group/condition, given as a discrete number and unit of measurement
☐	☒	A statement on whether measurements were taken from distinct samples or whether the same sample was measured repeatedly
☐	☒	The statistical test(s) used AND whether they are one- or two-sided<br>*Only common tests should be described solely by name; describe more complex techniques in the Methods section.*
☒	☐	A description of all covariates tested
☐	☒	A description of any assumptions or corrections, such as tests of normality and adjustment for multiple comparisons
☐	☒	A full description of the statistical parameters including central tendency (e.g. means) or other basic estimates (e.g. regression coefficient) AND variation (e.g. standard deviation) or associated estimates of uncertainty (e.g. confidence intervals)
☐	☒	For null hypothesis testing, the test statistic (e.g. *F*, *t*, *r*) with confidence intervals, effect sizes, degrees of freedom and *P* value noted<br>*Give P values as exact values whenever suitable.*
☒	☐	For Bayesian analysis, information on the choice of priors and Markov chain Monte Carlo settings
☒	☐	For hierarchical and complex designs, identification of the appropriate level for tests and full reporting of outcomes
☐	☒	Estimates of effect sizes (e.g. Cohen's *d*, Pearson's *r*), indicating how they were calculated

Our web collection on statistics for biologists contains articles on many of the points above.

Software and code

Policy information about availability of computer code

Data collection	Protein identities were quantified from LC-MS/MS spectra with MSGF+.<br>Reporter ion abundances were extracted from each spectrum using MASIC.
Data analysis	R version 4.1.1.<br>All available code for the complete analysis is found in Open Science Framework (https://osf.io/r8dbe/).

For manuscripts utilizing custom algorithms or software that are central to the research but not yet described in published literature, software must be made available to editors and reviewers. We strongly encourage code deposition in a community repository (e.g. GitHub). See the Nature Portfolio guidelines for submitting code & software for further information.

Data

Policy information about availability of data

All manuscripts must include a data availability statement. This statement should provide the following information, where applicable:

- Accession codes, unique identifiers, or web links for publicly available datasets
- A description of any restrictions on data availability
- For clinical datasets or third party data, please ensure that the statement adheres to our policy

Raw spectra from proteomics is publicly available in the MassIVE database (https://massive.ucsd.edu/ProteoSAFe/dataset.jsp?

task=46adedf2813f4598baffb6f764c46513) under subdirectory MSV000092155. The complete pipeline for conducting all analyses and figures, and the input data to generate these, can be found on Open Science Framework (https://osf.io/r8dbe/).

Research involving human participants, their data, or biological material

Policy information about studies with human participants or human data. See also policy information about sex, gender (identity/presentation), and sexual orientation and race, ethnicity and racism.

Reporting on sex and gender	No human subject data was used in this study.
Reporting on race, ethnicity, or other socially relevant groupings	No human subject data was used in this study.
Population characteristics	No human subject data was used in this study.
Recruitment	No human subject data was used in this study.
Ethics oversight	No human subject data was used in this study.

Note that full information on the approval of the study protocol must also be provided in the manuscript.

Field-specific reporting

Please select the one below that is the best fit for your research. If you are not sure, read the appropriate sections before making your selection.

☒ Life sciences ☐ Behavioural & social sciences ☐ Ecological, evolutionary & environmental sciences

For a reference copy of the document with all sections, see nature.com/documents/nr-reporting-summary-flat.pdf

Life sciences study design

All studies must disclose on these points even when the disclosure is negative.

Sample size	The 12 conditions were chosen for a full-factorial design with a goal of 3-4 (standard for the field) biological replicates for each condition, which was amenable for isobaric-tag labeling in proteomics. Sample size was determined sufficient for the statistical methods used based on PCA and the behaviors of known protein biomarkers for iron deficiency, algal lipid accumulation, and photosynthesis.
Data exclusions	As listed in methods, two proteomics samples (hxk1-1 –Fe +Glc, IDs: 37 and 40) showed strong evidence of incomplete isobaric tag labeling and were removed from statistical categorization.
Replication	All experimental batch replicates for growth curves and photosynthesis of the strain Open Science Framework (https://osf.io/r8dbe/). The html files of the R notebooks include experimental batch comparison figures of biomass and photosynthesis showing replication was successful for these parameters. For proteomics, replication was validated by PCA and autocorrelation, and enrichments for photosynthesis and lipid accumulation proteins were validated by their overlap with peer-reviewed studies.
Randomization	Cultures were picked randomly for nutrient treatments and were not labeled until treatments occurred.
Blinding	Blinding was not used and would have no effect on the outcome of data output from instruments or analyses.

Reporting for specific materials, systems and methods

We require information from authors about some types of materials, experimental systems and methods used in many studies. Here, indicate whether each material, system or method listed is relevant to your study. If you are not sure if a list item applies to your research, read the appropriate section before selecting a response.

Materials & experimental systems

n/a	Involved in the study	
☒	☐	Antibodies
☒	☐	Eukaryotic cell lines
☒	☐	Palaeontology and archaeology
☒	☐	Animals and other organisms
☒	☐	Clinical data
☒	☐	Dual use research of concern
☐	☒	Plants

Methods

n/a	Involved in the study	
☒	☐	ChIP-seq
☒	☐	Flow cytometry
☒	☐	MRI-based neuroimaging

Dual use research of concern

Policy information about dual use research of concern

Hazards

Could the accidental, deliberate or reckless misuse of agents or technologies generated in the work, or the application of information presented in the manuscript, pose a threat to:

No	Yes	
☒	☐	Public health
☒	☐	National security
☒	☐	Crops and/or livestock
☒	☐	Ecosystems
☒	☐	Any other significant area

Experiments of concern

Does the work involve any of these experiments of concern:

No	Yes	
☒	☐	Demonstrate how to render a vaccine ineffective
☒	☐	Confer resistance to therapeutically useful antibiotics or antiviral agents
☒	☐	Enhance the virulence of a pathogen or render a nonpathogen virulent
☒	☐	Increase transmissibility of a pathogen
☒	☐	Alter the host range of a pathogen
☒	☐	Enable evasion of diagnostic/detection modalities
☒	☐	Enable the weaponization of a biological agent or toxin
☒	☐	Any other potentially harmful combination of experiments and agents
